# Continuous ambulatory peritoneal dialysis-associated *Histoplasma capsulatum* peritonitis: a case report and literature review

**DOI:** 10.1186/s12879-020-05441-5

**Published:** 2020-09-29

**Authors:** Thanat Ounsinman, Piriyaporn Chongtrakool, Nasikarn Angkasekwinai

**Affiliations:** 1 Division of Infectious Diseases and Tropical Medicine, Department of Medicine, Faculty of Medicine Siriraj Hospital, Mahidol University, Bangkok, Thailand; 2 Department of Microbiology, Faculty of Medicine Siriraj Hospital, Mahidol University, Bangkok, Thailand

**Keywords:** *Histoplasma capsulatum*, Continuous ambulatory peritoneal dialysis, Fungal peritonitis, End-stage renal disease

## Abstract

**Background:**

Fungal peritonitis (FP) is a rare complication of peritoneal dialysis. We herein describe the second case in Asia of *Histoplasma capsulatum* peritonitis associated with continuous ambulatory peritoneal dialysis (CAPD).

**Case presentation:**

An 85-year-old woman with end-stage renal disease (ESRD) who had been on CAPD for 3 years and who had a history of 3 prior episodes of peritonitis presented with intermittent abdominal pain for 2 weeks and high-grade fever for 3 days. Elevated white blood cell (WBC) count and rare small oval budding yeasts were found in her peritoneal dialysis (PD) fluid. From this fluid, a white mold colony was observed macroscopically after 7 days of incubation, and numerous large, round with rough-walled tuberculate macroconidia along with small smooth-walled microconidia were observed microscopically upon tease slide preparation, which is consistent with *H. capsulatum.* The peritoneal dialysis (PD) catheter was then removed, and it also grew *H. capsulatum* after 20 days of incubation. The patient was switched from CAPD to hemodialysis. The patient was successfully treated with intravenous amphotericin B deoxycholate (AmBD) for 2 weeks, followed by oral itraconazole for 6 months with satisfactory result. The patient remains on hemodialysis and continues to be clinically stable.

**Conclusion:**

*H. capsulatum* peritonitis is an extremely rare condition that is associated with high morbidity and mortality. Demonstration of small yeasts upon staining of PD fluid, and isolation of slow growing mold in the culture of clinical specimen should provide important clues for diagnosis of *H. capsulatum* peritonitis. Prompt removal of the PD catheter and empirical treatment with amphotericin B or itraconazole is recommended until the culture results are known.

## Background

Fungal peritonitis (FP) is a rare and serious complication of chronic peritoneal dialysis (PD), and it is associated with a high rate of hospitalization, catheter removal, permanent transfer to hemodialysis, and death [1]. Although FP is primarily suspected in patients with prior antibiotic use within 3 months [2, 3], early diagnosis of FP still remains challenging since it is difficult to distinguish from bacterial peritonitis. *Candida* spp. was the predominant pathogen causing FP in many previous studies [4]. FP caused by fungi other than *Candida spp.* is only rarely reported. Herein, we report a case and literature review of fungal peritonitis caused by *Histoplasma capsulatum* that presented with fever and subacute-onset of abdominal pain.

## Case presentation

An 85-year-old woman with end-stage renal disease (ESRD) presented with abdominal discomfort and high-grade fever for 2 days. She was diagnosed ESRD in 2017, and has been treated with peritoneal dialysis (PD) since then. Two weeks after catheter insertion, she had difficulty draining infused dialysate fluid due to catheter malposition, which required repositioning of Tenckhoff catheter owing to partial catheter obstruction from a fibrin clot. Since that revision, she has been on continuous ambulatory peritoneal dialysis (CAPD) without further mechanical complication. However, there were 3 episodes of CAPD-related infection that occurred within 6 months of her most recent admission.

The first episode started in August 2019 when she had cloudy PD fluid effluent without fever or abdominal complaint. Peritoneal fluid analysis showed white blood cell count (WBC) of 268 cells/mm^3^, with 55% neutrophils, 37% lymphocytes, and 8% eosinophils. Culture-negative PD-associated peritonitis was diagnosed since blood and PD fluid cultures for aerobic bacteria reported no growth. She was successfully treated with intraperitoneal cefazolin and ceftazidime for 14 days. The second episode occurred in September 2019. Due to recurrent catheter malposition from omental wrapping, she underwent a second revision of her PD catheter. After the operation, she had a high-grade fever with non-specific organ symptoms. PD fluid analysis showed a WBC count of 103 cells/mm^3^, with 65% neutrophils, 21% lymphocytes, and 10% eosinophils. Blood and PD fluid culture for aerobic bacteria were no growth. Computed tomography (CT) of the whole abdomen revealed no intra-abdominal collection, and that the tip of the PD catheter was in the proper position. She then gradually improved with a reduction of WBC in PD fluid to 7 cells/mm^3^ after treatment with intraperitoneal cefazolin and ceftazidime for 14 days. The third episode occurred near the end of December 2019 when she presented with a high-grade fever, nausea, and vomiting, but without any remarkable physical findings. Two sets of blood culture revealed alpha-hemolytic streptococci. PD fluid analysis revealed a WBC count of 14 cells/mm^3^ without differential count due to a low cell count, and culture revealed a rare colony of alpha-hemolytic streptococci. Streptococcal PD-associated peritonitis and septicemia were diagnosed, and she was successfully treated with intravenous ceftriaxone for 14 days. Fungal culture of PD fluid was not sent during any of the first three episodes of culture negative peritonitis.

In January 2020 or 2 weeks prior to her most recent admission, she was complaining of intermittent abdominal pain during drainage of dialysate fluid from her abdomen and a low-grade fever for 3 days. Her PD fluid effluent had also become cloudy. She was febrile with a temperature of 38.2 ͦ C, but reported no abdominal tenderness on physical examination. PD fluid analysis revealed a WBC count of 380 cells/mm^3^, with 86% neutrophils, 11% lymphocytes, and 1% eosinophils. She was then empirically treated with intraperitoneal vancomycin and ceftazidime. Gram stain of PD fluid revealed few polymorphonuclear cells with no observed bacteria; however, small oval budding yeasts were demonstrated. PD fluid culture for bacteria was sent. The culture for fungus was first sent after recurrent peritonitis that occurred within the past 5 months. Therefore, oral fluconazole 100 mg/day was given concurrently with intraperitoneal antibiotic administration. After intraperitoneal antibiotic and fluconazole treatment for one week, her fever and abdominal pain were gradually improved. Peritoneal fluid analysis showed a marked decrease in white cell count (1 cell/mm^3^), culture of dialysis fluid showed no evidence of bacterial growth, and fungal culture was still pending.

Despite treatment with intraperitoneal antibiotic and oral fluconazole 100 mg daily for 2 weeks, she developed recurrent abdominal discomfort, loss of appetite, cloudy PD effluent for 3 days, and high-grade fever for 1 day prior to admission. She was then admitted on 24 January 2020. On examination, her body temperature was 38.4 °C, and generalized abdominal tenderness was found. All other examination findings, including investigation for PD catheter exit site infection, were unremarkable.

**Fig. 1:**
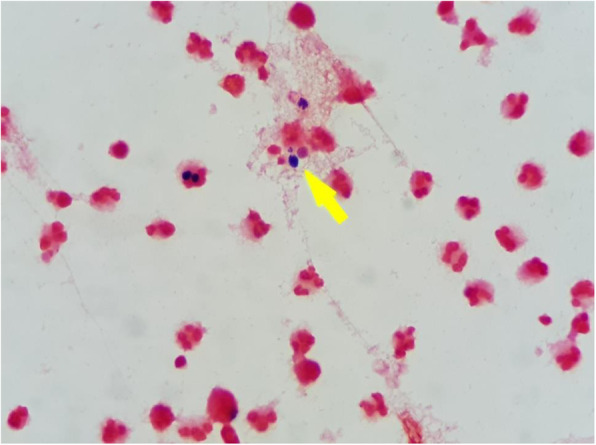
Gram stain finding of peritoneal dialysis fluid demonstrated rare intracellular oval budding yeast

**Fig. 2:**
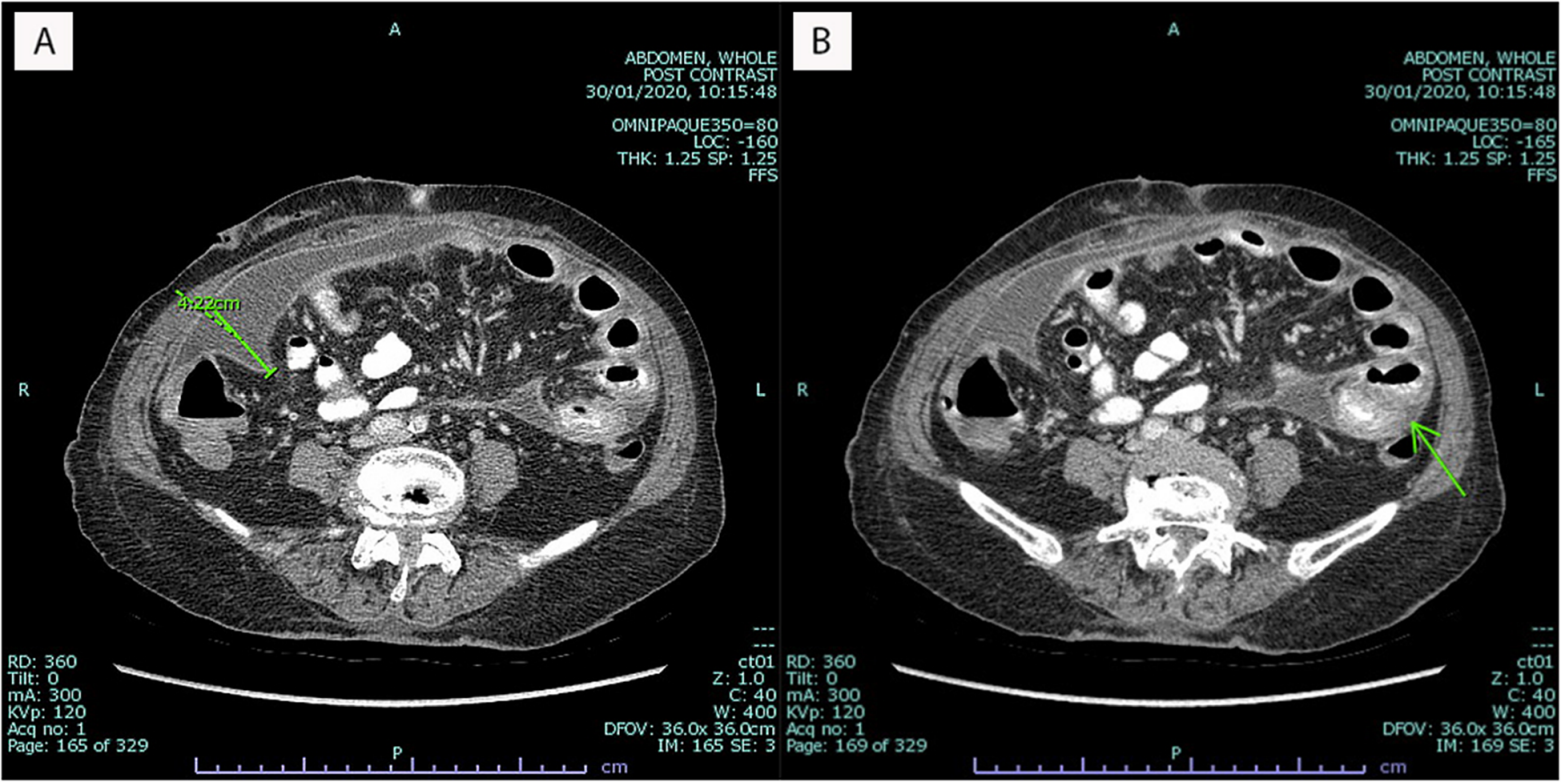
Computed tomography of whole abdomen. **a** Diffuse smooth thickened enhancing peritoneum with moderate amount of turbid ascites along the mesentery and in the pelvic cavity. **b** Localized fixation of small bowel loops within the thickened peritoneum

Initial laboratory investigations of complete blood count (CBC) revealed a WBC count of 11,340/μL, with 83.5% neutrophils, 9.5% lymphocytes, and 0.2% eosinophils; a hemoglobin level of 9.9 g/dL; and, a platelet count of 295,000 /μL. Blood chemistry showed a high level of creatinine, which was consistent with her renal disease. Analysis of her PD fluid effluent showed an elevated WBC count of 278 cells/mm^3^, with 92% neutrophils, 8% lymphocytes, and rare red blood cells (RBC). Gram stain of the dialysate effluent still demonstrated rare small oval budding yeasts (Fig. 1). A CT scan of her abdomen revealed diffuse smooth thickened enhancing peritoneum with a moderate amount of turbid ascites (18–21 HU) along the mesentery and in the pelvic cavity. Localized fixation of small bowel loops within the thickened peritoneum was also found, which is suggestive of early sclerosing encapsulating peritonitis (Fig. 2).

**Fig. 3:**
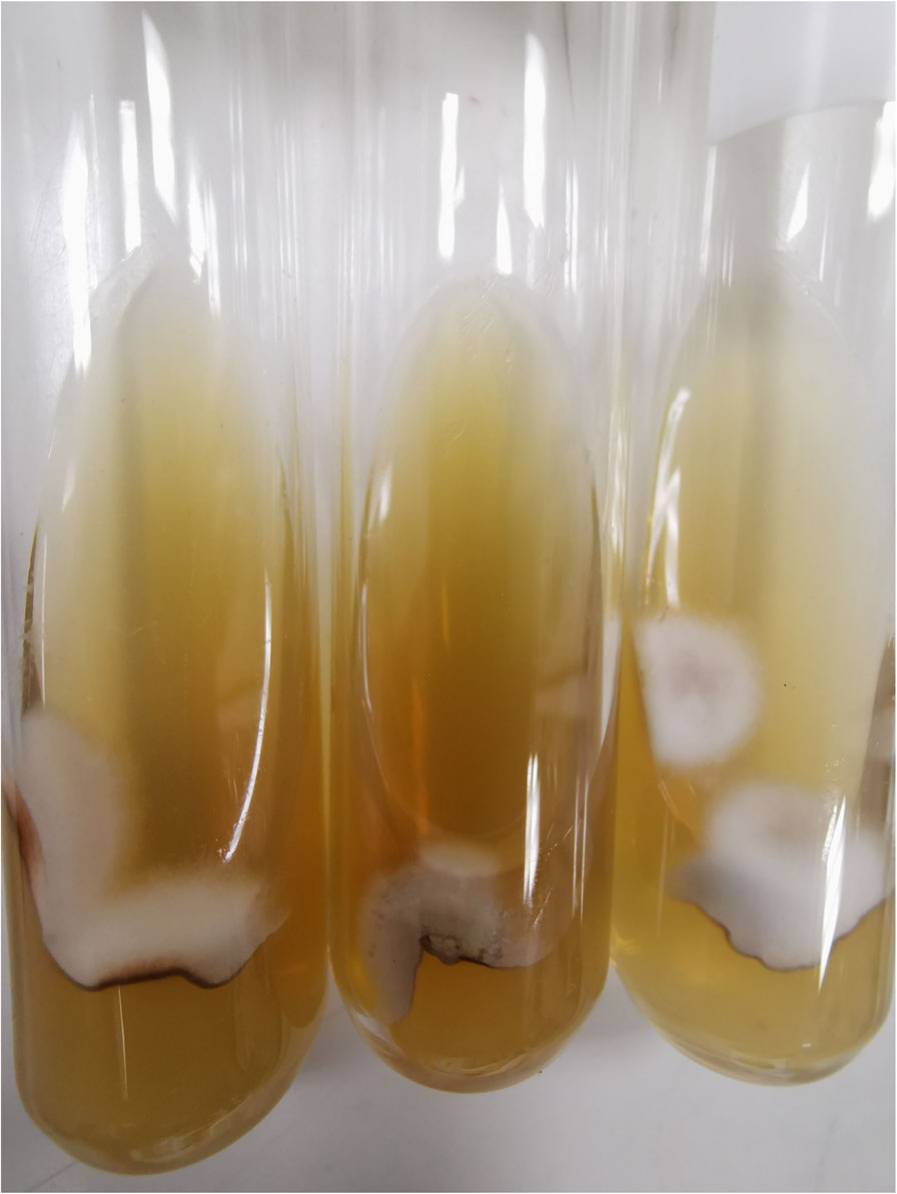
Fungal culture on Sabouraud dextrose agar demonstrated a white colony of mold

**Fig. 4:**
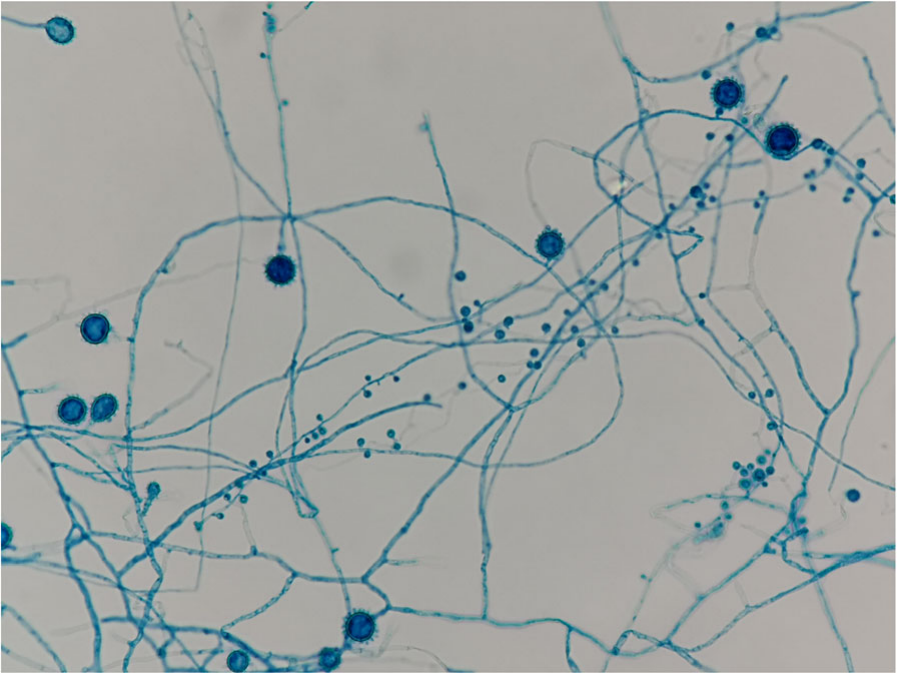
Microscopic morphology demonstrated large, round with rough-walled tuberculate macroconidia and small round-to-oval smooth-walled microconidia of *Histoplasma capsulatum*

She was empirically treated with intravenous meropenem 500 mg/day, vancomycin 500 mg every 72 h, and amphotericin B deoxycholate (AmBD) 0.7 mg/kg/day. The Tenckhoff catheter was removed on the day after admission due to suspected fungal peritonitis. Intraoperative finding revealed turbid dialysate fluid and fibrotic scar at the exit site of the peritoneal dialysis catheter. Renal replacement therapy was switched to intermittent hemodialysis via a newly inserted temporary double-lumen catheter at the right internal jugular vein. Bacterial culture reported no growth thereafter. After two weeks of hemodialysis, a white colony of mold was observed macroscopically from a fungal culture taken two weeks prior to admission (Fig. 3). Microscopic morphology from PD fluid culture demonstrated numerous large, rounded with rough-walled tuberculate macroconidia along with small smooth-walled microconidia, which is consistent with *H. capsulatum* (Fig. 4). Another specimen of PD fluid also demonstrated the same pathogen.

The final diagnosis was CAPD-associated *H. capsulatum* peritonitis. Intravenous AmBD at a dose of 0.7 mg/kg/day was continued for a total of 2 weeks, followed by oral itraconazole capsule 600 mg daily for 3 days, and 200 mg twice daily thereafter. Our patient’s abdominal pain and fever were gradually improved. She was discharged and subsequently given a course of oral itraconazole 200 mg twice daily for a total duration of 6 months. The patient was continued on hemodialysis and is currently stable. No adverse event of itraconazole was observed during follow-up.

## Discussion

CAPD-associated *H. capsulatum* peritonitis is an extremely rare infectious complication in chronic PD patients. Approximately 3–6% of all peritonitis episodes in patients undergoing PD were caused by fungus, and *Candida* spp. is the most often reported group of fungal pathogens [3]. Similarly, nationwide surveillance in Thailand during 2009 to 2010 found FP in approximately 6% of culture-positive peritonitis in CAPD patients [5]. Reported complications of FP in CAPD included inability to continue CAPD, longer length of hospitalization, and high mortality [6].

Due to the rarity of this case, we performed systematic literature searches of the Medline databases for articles published from 1985 to 2020. No language restriction was applied and reference lists of all included studies were manually searched for other potentially eligible studies. Our search results revealed that no cohort or case control studies of CAPD-associated *H. capsulatum* peritonitis have been published. We identified only 7 case reports of *H. capsulatum* peritonitis in CAPD patients published over the past three decades (Table 1). Most cases were reported from endemic region of *Histoplasma* spp*.* or areas where there had been an outbreak of histoplasmosis, as follows: 4 cases from the United States [7–10], and 2 cases from Brazil [11, 12]. The one case reported from Asia was reported from Hong Kong [13]. However, sporadic cases of histoplasmosis have been reported throughout the world, especially in AIDS patients [14]. We herein report the second case in Asia of isolated *H. capsulatum* peritonitis in a CAPD patient.

**Table 1:** Literature review for previously reported cases of CAPD-associated *Histoplasma capsulatum* peritonitis

No.	Reported year, country	Age/ gender	Duration of CAPD	Previous BP (No.)	Clinical presentation	PD fluid analysis	Catheter removal	Treatment	Reference
1	2018, USA	63/F	4 years	Yes (2)	Abdominal pain, fever	WBC 2173 cells/mm^3^, 96% Ne	No	NA	[7]
2	2012, USA	75/M	> 2 years	Yes (2)	Abdominal pain, fever	WBC 562 cells/mm^3^	Yes	ITZ for 1 year	[8]
3	2010, USA	62/M	4 years	No	Abdominal pain, no fever	WBC 180 cells/mm^3^, 41% Ne	Yes	ITZ for 6 months	[9]
4	2006, USA	27/F	NA	NA	Cloudy PD	WBC 377 cells/mm^3^, 88% Ne	Yes	AmBD for 2 weeks and ITZ for 2 months	[10]
5	1994, Brazil	64/F	4 months	No (1)	Abdominal pain, cloudy PD	WBC 2133 cells/mm^3^, 95% Ne	Yes	AmBD, unknown duration	[11]
6	1993, Brazil	50/F	2 years	NA	Abdominal pain, fever	NA	Yes	AmBD, unknown duration	[12]
7	1991, Hong Kong	46/M	2 years	Yes (1)	Abdominal pain, fever	WBC 370 cells/mm^3^	No	FCZ, 5-FC, AmBD, unknown duration	[13]

*Abbreviations*: *AmBD* amphotericin B, *BP* bacterial peritonitis, *CAPD* continuous ambulatory peritoneal dialysis, *F* female, *FCZ* fluconazole, *ITZ* itraconazole, *M* male, *Ne* neutrophil, *No*. number, *PD* peritoneal dialysis, *WBC* white blood cell, *5-FC* flucytosine, *USA* United States of America, *NA* not available

Diagnosis of *H. capsulatum* peritonitis requires a high degree of suspicion. In addition to it being a rare disease, a lack of specific symptoms and signs make it’s difficult to differentiate from bacterial peritonitis. Previous episode of bacterial peritonitis and failure to respond to initial empirical antibiotic treatment for common bacteria should prompt the attending physician to consider a diagnosis of infection with unusual organisms [3]. Microbiological laboratory testing can also help to facilitate an early diagnosis of histoplasmosis. *H. capsulatum* stains poorly with Gram stain, so it is rarely detected by this method. Calcofluor white, which is a fluorescent stain that binds chitin in the cell wall of all fungi, is useful for identifying *H. capsulatum* in clinical specimens. Careful examination of special staining of PD fluid may, thus, help in making a provisional diagnosis of *H. capsulatum* peritonitis. Among the 7 cases shown in Table 1, the WBC count in PD fluid varied from 180 to 2173 cells/mm^3^. Isolation of *H. capsulatum* remains the gold standard for diagnosis of histoplasmosis. When incubated on appropriate medium at 25 to 30 °C, growth of the mycelial phase occurs most commonly within 2 to 3 weeks, but may take up to 8 weeks [15]. If positive, characteristic large with rough-walled tuberculate macroconidia (7 to 15 μm in size) and small smooth-walled microconidia (2 to 6 μm in size) will be demonstrated.

According to guidelines issued by the International Society for Peritoneal Dialysis (ISPD) in 2016, the administration of antifungal agents and immediate catheter removal are recommended when fungi are identified in PD effluent [16]. Both interventions lower the risk of recurrent fungal peritonitis and death [6]. The PD catheter was removed in 5 of the 7 cases in previous reports. Intravenous AmBD has poor peritoneal bioavailability and flucytosine is not widely available, so other agents, including azole drugs and echinocandin, have been frequently used to treat *Candida* spp. and filamentous fungi [16]. However, intravenous AmBD and itraconazole are listed as recommended drugs for treatment of histoplasmosis. No specific recommendation was provided for the duration of treatment of *H. capsulatum* peritonitis [17]. Most previous reports described 6–12 months of oral itraconazole with or without initial intravenous AmBD or flucytosine [7–10]. Our patient received 2 weeks of AmBD followed by 6 months of oral itraconazole, and she had a good response to treatment.

In conclusion, *H. capsulatum* peritonitis is an extremely rare condition that is associated with high morbidity and mortality. Demonstration of small yeasts upon staining of PD fluid, and isolation of slow growing mold in the culture of clinical specimen should provide important clues for diagnosis of *H. capsulatum* peritonitis. Prompt removal of the PD catheter and empirical treatment with amphotericin B or itraconazole is recommended until the culture results are known.

## Data Availability

All data generated or analysed during this study are included in this published article.

## Abbreviations

AmBD: Amphotericin B deoxycholate
CAPD: Continuous ambulatory peritoneal dialysis
CBC: Complete blood count
CT: Computed tomography
ESRD: End-stage renal disease
FP: Fungal peritonitis
HR: Heart rate
HU: Hounsfield unit
PD: Peritoneal dialysis
RBC: Red blood cell
WBC: White blood cell
